# Sliding Amyand’s hernia: a case report and review of literature

**DOI:** 10.1093/jscr/rjab288

**Published:** 2021-07-05

**Authors:** Amr Elgazar, Ahmed K Awad, Debvarsha Mandal, Raid M Faddah, Zachary Elder, Sheref A Elseidy

**Affiliations:** Department of General Surgery, Ain Shams University, Cairo, Egypt; Department of General Surgery, Ain Shams University, Cairo, Egypt; Caribbean Medical University, Chicago, IL, USA; Detroit Medical Center, Heart and Vascular Institute, Detroit, Michigan, USA; American University of the Caribbean School of Medicine, USA; Department of Cardiovascular Diseases, Ain Shams University, Cairo, Egypt

**Keywords:** Hernia, Amyand’s hernia, Inguinal hernia, case report

## Abstract

First operated by Claudius Amyand in 1735. Amyand’s hernia is a rare presentation and accounts for only 1% of all inguinal hernias. Amyand’s hernia is described when the appendix is trapped within an inguinal hernia. In most cases, Amyand’s hernia is an incidental finding intra-operatively due to variable clinical manifestations, and features. Amyand’s hernia has variable theories explaining its pathophysiology besides having multiple proposed surgical approaches either via laparoscopic or open repair and with the latter being in a debate of pro and against mesh repair. We present a case of a sliding Amyand’s hernia in which the vermiform appendix and part of the cecum were adherents to the wall of a right inguinal hernial sac. Amyand’s hernia is a rare form of inguinal hernias and its presentation is widely variable. However, in most cases, it is non-complicated and is found as an incidental intraoperative finding. Many studies debate among different diagnostic and management approaches to serve a better outcome with fewer operative complications.

## INTRODUCTION

Hernia is a protrusion of an organ or its fascia through the wall of a containing cavity [1]. When an organ is contained within a non-reducible hernia, it is termed an incarcerated hernia. While a strangulated hernia is when the blood supply to the edematous, incarcerated bowel is compromised secondary to venous and lymphatic obstruction, Amyand’s hernia is when the appendix is trapped within the inguinal hernia. Amyand’s hernia is a rare presentation and accounts for only 1% of all inguinal hernias [1]. In most cases, Amyand’s hernia is an incidental finding intra-operatively due to non-specific clinical manifestations and various clinical features. Moreover, as the clinical presentations differ from one condition to another, surgical approaches vary accordingly to the condition in which the vermiform appendix is present [2].

## CASE PRESENTATION

A 47-year-old male patient presented to our outpatient clinic complaining of 4 years duration of right groin swelling. No associated comorbidities were found. On examination inspection of the swelling, it was about 5 × 7 cm, oval in shape, soft inconsistency, with well-defined edges and smooth surface and gives an expansible impulse on coughing. On scrotal neck test, it was inguinoscrotal (funicular type). The hernia was completely reducible with no signs of obstruction or strangulation. The patient was scheduled for open hernia repair.

**Figure 1:**
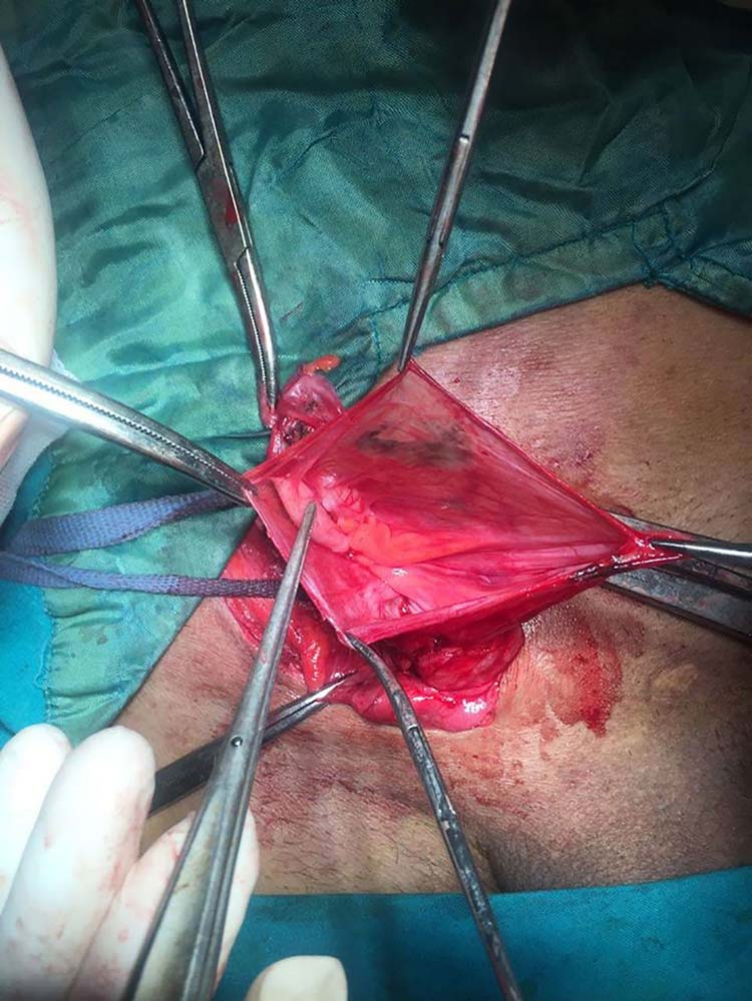
Sliding Amyand’s hernia.

Routine pre-operative laboratory workup was done including TLC, ESR and CRP showed no abnormalities. Intra-operatively, the hernial sac was identified and dissected from the spermatic cord down to the internal ring; upon opening the hernia sac, a vermiform appendix with its mesoappendix and part of the cecum were completely adherent to the wall of the sac Fig. 1. The appendix was not inflamed and the decision of sac closure and reduction into the abdominal cavity through the internal ring with performing the usual Liechtenstein tension-free hernioplasty was made.

The post-operative course was uneventful and the patient was discharged from the hospital on next day. Follow-up examinations and imaging showed no complications.

## DISCUSSION

Claudius Amyand was the first to operate and report a case of inguinal hernia harboring a vermiform appendix in 1735 [3]. The incidence of Amyand’s hernia is rarely ranging from 0.19 to 1.7%. Appendicitis in inguinal hernia is rarer and range from 0.07 to 0.13%. Perforated appendix within the inguinal hernia is less common and it accounts for only 0.1% of all cases of appendicitis [4–6]. Most of Amyand’s hernia occurs on the right side due to the normal anatomical position of the appendix; however, left-sided Amyand’s hernias have been reported [6]. Johnson *et al.* suggested that appendicitis in such cases could be due to extraluminal compression rather than intraluminal obstruction; several theories on the pathophysiology of this rare disorder have been reported however an exact cause is still unclear [7].

Losanoff and Basson classify the Amyand’s hernia into four types according to the condition of the vermiform appendix; Type 1: Normal appendix within inguinal hernia; Type 2: Acute appendicitis within inguinal hernia with no abdominal sepsis; Type 3: Acute appendicitis within the inguinal hernia, abdominal wall or peritoneal sepsis; Type 4: Acute appendicitis within an inguinal hernia, related or unrelated abdominal pathology [8]. Rikki modified this classification and add type 5 to it which included incisional and it is subdivided into (5A): Normal appendix within an incisional hernia; (5B): Acute appendicitis within an incisional hernia, no abdominal sepsis, and (5C): Acute appendicitis within an incisional hernia, abdominal wall or peritoneal sepsis or with previous surgery [9].

Imaging studies are seldom requested to diagnose inguinal hernias and diagnosis depends primarily on clinical examination especially in completely reducible uncomplicated cases. Ultrasound can detect vermiform appendix within the hernia sac [10]. Computed tomography (CT) scan with contrast is more specific and sensitive than ultrasound especially in complicated cases [11]. All of this can guide the pre-operative diagnosis; however, the final management is undertaken on an intra-operative basis. With mortality ranging from 14 to 30% and mostly owed to the peritoneal spread of sepsis, Amyand’s hernia requires proper surgical management—surgery is both therapeutic and diagnostic, yet the definitive surgical management is still unclear.

While many authors do not recommend mesh repair in cases where there is acute appendicitis and appendectomy is needed to be performed, others see that it is safe and it could reduce the rate of recurrence of inguinal hernia even in the presence of a septic environment [12]. Ivashchuk *et al.* recommended that the presence of appendicitis and hence appendectomy should not limit the use of mesh in the repair and the best management option should be according to surgical preference [2]. Laparoscopic management of Amyand’s hernia has also been proposed as when Rehman *et al.* and Vermillion *et al.* were the first to use laparoscopy in Amyand’s hernia management reporting low septic environment, better outcomes and better recovery [13, 14]. The best management strategy for Amyand’s hernia is debatable, yet its answer lay behind these questions: is appendicectomy required? which approach should be used? What form of repair should be undertaken?

In our case, the appendix and part of the cecum were adherents to the hernia sac wall. The appendix was not inflamed—type1—therefore not requiring appendectomy. Mesh repair was done without evidence of recurrence of hernia at 1-year follow-up. Green and Gutwein reported the only similar case to ours: a type 1 Amyand’s hernia to which herniotomy with hernioplasty was performed without the need of appendectomy [15]. On the other side, Shamim reported a case in a 27-year-old male in which the appendix—7.5 cm in length—was a sliding component of the sac but not adherent to the wall. Although he performed appendectomy as it looked inflamed with mesh repair, the appendix was normal in specimen final histopathology [16].

## CONCLUSION

Amyand’s hernia is a rare entity and its presentation differs from case to case. The diagnosis is very difficult owed due to the uncomplicated presentations which are mostly seen in these patients. Management modalities are debatable and provide many plausible and rationally accepted pros and cons that steer the approach toward one surgical modality versus the other. However, in our opinion, open repair with mesh without the need for an appendectomy should be the definitive strategy in absence of inflammation, sepsis, provided by a clean intra-operative environment and skilled surgical expertise.

## References

[1] Ali SM, Malik KA, Al-Qadhi H. Amyand’s hernia: study of four cases and literature review. Sultan Qaboos Univ Med J 2012;12:232–6. 22548145 10.12816/0003119 PMC3327573

[2] Ivashchuk G, Cesmebasi A, Sorenson EP, Blaak C, Tubbs SR, Loukas M. Amyand’s hernia: a review. Med Sci Mon Int Med J Exp Clin Res 2014;20:140. 10.12659/MSM.889873 PMC3915004 24473371

[3] Amyand CVIII . Of an inguinal rupture, with a pin in the appendix coeci, incrusted with stone; and some observations on wounds in the guts. Philos Trans R Soc Lond 1736;39:329–42.

[4] Sharma H, Gupta A, Shekhawat NS, Memon B, Memon MA. Amyand’s hernia: a report of 18 consecutive patients over a 15-year period. Hernia 2007;11:31–5. 17001453 10.1007/s10029-006-0153-8

[5] Häuser U, Merkle P. Differential diagnosis of incarcerated inguinal hernia in an infant: appendicitis in the hernial sac. Z Kinderchir 1984;39:72–3. 6730710 10.1055/s-2008-1044176

[6] Pellegrino JM, Feldman SD. Case report: acute appendicitis in an inguinal hernia. N J Med J Med Soc N J 1992;89:225–6. 1574205

[7] Johnson CD . Appendicitis in external hernia. Ann R Coll Surg Engl 1982;64:283. PMC2494146 19310832

[8] Losanoff JE, Basson MD. Amyand hernia: a classification to improve management. Hernia 2008;12:325–6. 18214637 10.1007/s10029-008-0331-y

[9] Singal R, Mittal A, Gupta A, Gupta S, Sahu P, Sekhon MS. An incarcerated appendix: report of three cases and a review of the literature. Hernia 2012;16:91–7. 20740297 10.1007/s10029-010-0715-7

[10] Mebis W, Hoste P, Jager T. Amyand’s Hernia. J Belg Soc Radiol 2018;102:8. 10.5334/jbr-btr.1402 PMC6095048 30128422

[11] Ivashchuk G, Cesmebasi A, Sorenson EP, Blaak C, Tubbs SR, Loukas M. Amyand’s hernia: a review. Med Sci Monit Int Med J Exp Clin Res 2014;20:140. 10.12659/MSM.889873 PMC3915004 24473371

[12] Chatzimavroudis G, Papaziogas B, Koutelidakis I, Tsiaousis P, Kalogirou T, Atmatzidis S, et al. The role of prosthetic repair in the treatment of an incarcerated recurrent inguinal hernia with acute appendicitis (inflamed Amyand's hernia). Hernia 2009;13:335. 19418014 10.1007/s10029-009-0505-2

[13] Vermillion JM, Abernathy SW, Snyder SK. Laparoscopic reduction of Amyand's hernia. Hernia 1999;3:159–60.

[14] Rehman MR, Panteli C, Tsang T. Laparoscopic repair of Amyand’s hernia in an 8-week-old infant. Hernia 2010;14:443–5. 19806421 10.1007/s10029-009-0569-z

[15] Green J, Gutwein LG. Amyand's hernia: a rare inguinal hernia. J Surg Case Rep 2013;2013:rjt043. 24963899 10.1093/jscr/rjt043 PMC3813824

[16] Shamim M . Amyand’s hernia as a sliding component of inguinal hernia. J Surg Pak (Int) 2010;15:60.

